# Supplementary material for: Structural transitions in the GTP cap visualized by cryo-electron microscopy of catalytically inactive microtubules
Source: Proc Natl Acad Sci U S A. 2022 Jan 7;119(2):e2114994119. doi: 10.1073/pnas.2114994119 (PMC8764682; doi:10.1073/pnas.2114994119)
Supplement: Supplementary File [file pnas.2114994119.sapp.pdf]

PNAS
www.pnas.org

Supplementary Information for

Structural transitions in the GTP cap visualized by cryo-EM of catalytically inactive microtubules

Benjamin J LaFrance, Johanna Roostalu, Gil Henkin, Basil J Greber, Rui Zhang, Davide Normanno, Chloe McCollum, Thomas Surrey, Eva Nogales

Corresponding Authors: Thomas Surrey, Eva Nogales
Email: thomas.surrey@crg.eu, enogales@lbl.gov

This PDF file includes:

Figures S1 to S3
Table S1

Other supplementary materials for this manuscript include the following:

Movie S1 to S4

(A)

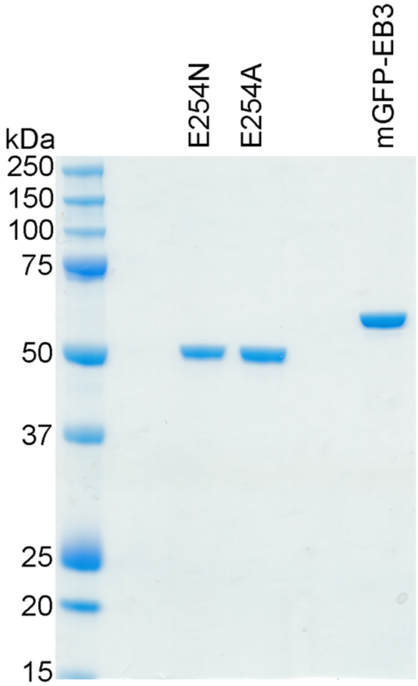

(B)

E254N nucleotide content		
	**% GDP**	**% GTP**
**exp. 1**	12.1	87.9
**exp. 2**	16.6	83.4
**exp. 3**	14.9	85.1
**Average**	**14.5**	**85.5**
**STDEV**	**2.3**	**2.3**

Figure S1. Biochemical characterization of mutant tubulin.
(A) Coomassie-stained SDS gel showing E254N tubulin, E254A tubulin, and mGFP-EB3 used for TIRF microscopy. **(B)** Table showing the detected GDP and GTP content of E254N MTs as obtained in three independent experiments by HPLC. The detected GTP content is similar to that of E254A MTs (1).

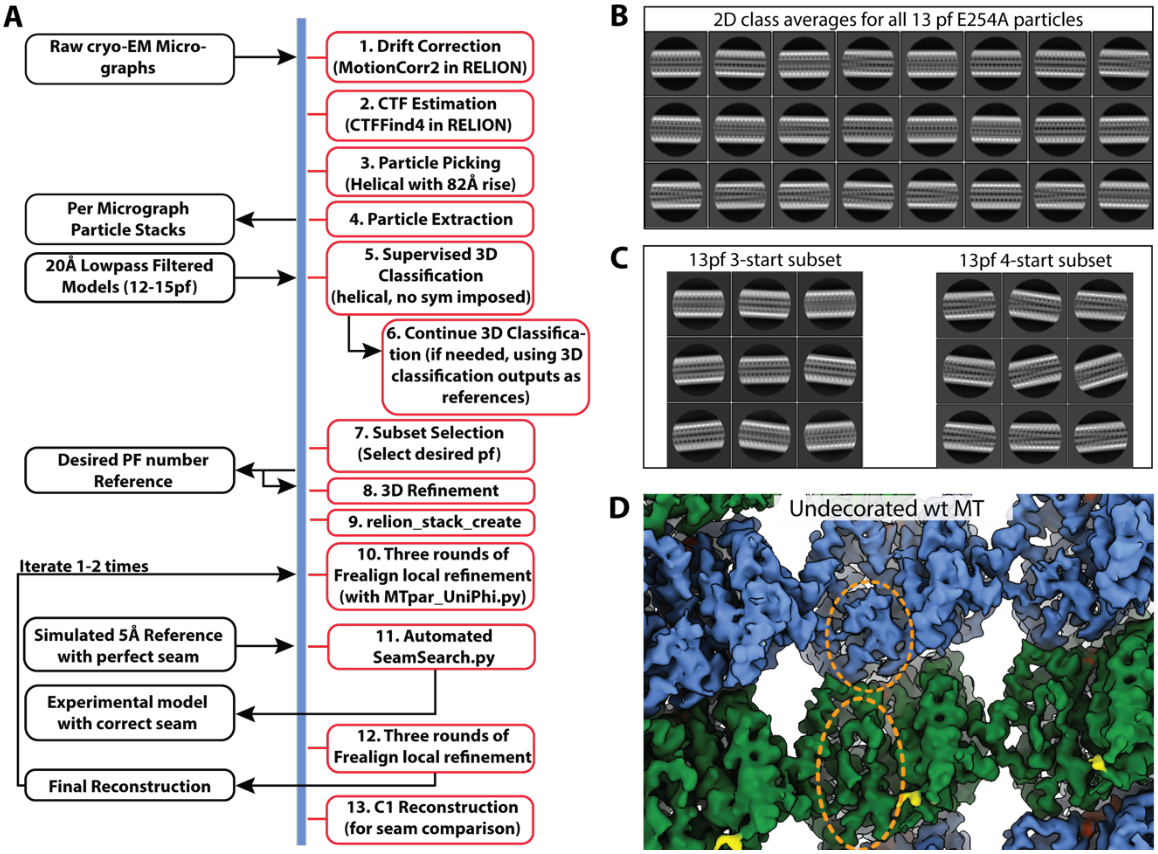

Figure S2. Cryo-EM data processing pipeline with example classes and structure.
(A) Schematic of the data processing pipeline used to reconstruct MT structures based on a hybrid approach between the recently described MIRP protocol (2) and the SeamSearch technique (3). Steps 1-9 are performed within the RELION framework. In order to resolve the 3-start and 4-start structures for E254A, the output from the first round of supervised 3D classification, along with the original references, were used to subclassify 13pf models. This was done 3 times, until no residual classification was observed. Furthermore, because the dataset corresponding to undecorated MTs (without an associated protein such as EB3 to serve as fiducial for the tubulin dimer), SeamSearch was necessary to separate α- and β-tubulin, as outlined in steps 10-12. **(B)** Initial 2D classification results obtained from the E254A dataset. **(C)** After classification based on protofilament type, an additional sub-classification for 13pf MTs to separate dimer twist revealed both “straight” classes (pfs running parallel to the MT axis in the 3-start lattice, left) and “super twisted” classes (corresponding to the 4-start lattice, right). **(D)** Structure of wildtype recombinant MTs and separation of α- and β-tubulin for undecorated MTs. The small region highlighted in yellow corresponds to additional density in the recombinant MTs that can be assigned to the internal His_6-tag present in β-tubulin. The distinction of α- and β-tubulin subunits (green and blue, respectively) obtained with our image analysis scheme in the absence of decoration with other factors is highlighted by the dashed orange ovals marking a distinctly longer loop in α-tubulin facing the MT lumen.

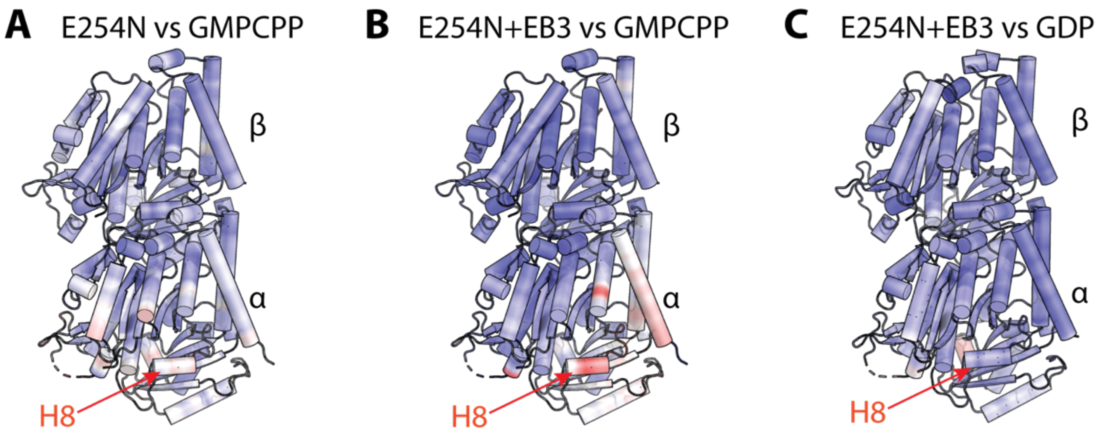

Figure S3. Mutant dimer structure versus the GMPCPP and GDP states within the MT. Displacement values for the tubulin dimer from various MT structures after alignment to β-tubulin and displayed after normalization to the same scale for all models (see Materials and Methods, blue-to-red coloring indicates 0Å-1Å displacement, respectively). The location of helix 8 (H8) which has been previously shown to have the greatest displacement up compaction (4) is indicated by the red arrow in each panel. This figure shows that in addition to the lattice parameters reported in Data Table 1, E254N resembles the GMPCPP state at the tubulin dimer structural level (A). Once bound by EB3, and accompanying lattice compaction, the EB3+E245N tubulin structure deviates from the GMPCPP MT structure (B) and very closely resembles that seen in the GDP MT (C).

Table S1. Data collection, 3D reconstruction, and refinement statistics.

**Dataset**	**wt**	**wt + kinesin**	**E254A (3/4)**	**EB3+E254A**	**E254N**	**E254N+EB3**
Microscope	Titan Krios	Titan Krios	Titan Krios	Titan Krios	Titan Krios	Titan Krios
Stage type	Autoloader	Autoloader	Autoloader	Autoloader	Autoloader	Autoloader
Voltage (kV)	300	300	300	300	300	300
Detector	Gatan K2	Gatan K2	Gatan K3	Gatan K3	Gatan K3	Gatan K3
Data Collection Software	SerialEM	SerialEM	SerialEM	SerialEM	SerialEM	SerialEM
Acquisition mode	Super-res	Super-res	Super-res	Super-res	Super-res	Super-res
Physical pixel size (Å)	0.575	0.460	0.595	0.460	0.575	0.575
Defocus range (µm)	0.5-2.5	0.7-2.4	0.6-2.5	0.7-2.5	1.0-2.7	1.0-2.7
Electron exposure (e^-/A^2)	40	40	40	40	40	40
**Reconstruction**	**EMD-25156**	**EMD-25157**	**EMD-25178**	**EMD-25159**	**EMD-25160**	**EMD-25161**
Session	18Dec07b	18Dec07c	19Jun03	20Sep10	20Feb03	20Feb04
Software	RELION 3.1 & Frealign	RELION 3.1 & Frealign	RELION 3.1 & Frealign	RELION 3.1 & Frealign	RELION 3.1 & Frealign	RELION 3.1 & Frealign
Particles picked	33,575	39,703	165,039	77,608	77,703	6,465
Particles final (13pf)	19,365	23,264	3-start: 68000<br>4-start: 26022	56,705	13,706	3,825
Extraction box size (pixels)	512^3	512^3	512^3	512^3	512^3	256^3
Final pixel size (Å)	0.92	0.92	1.19	0.92	1.15	2.30
Map resolution (Sym; Å)	3.8	3.6	3-start: 3.4<br>4-start: 3.7	3.5	3.8	5.0
Map sharpening B-factor ($Å^2$)	-88	-92	3-start: -38<br>4-start: -50	-72	-36	-100
**Coordinate refinement**						
Software	PHENIX	PHENIX	--	PHENIX	PHENIX	--
Refinement algorithm	REAL SPACE	REAL SPACE	--	REAL SPACE	REAL SPACE	--
Resolution cutoff (Å)	3.8	3.8	--	3.7	3.8	--
$FSC_{model\text{-}vs\text{-}map}$=0.5 (Å)	3.9	4.0	--	3.6	4.2	--
**Model**	**PDB-7SJ7**	**PDB-7SJ8**	**--**	**PDB-7SJ9**	**PDB-7SJA**	**--**
Number of residues	864	864	--	995	861	--
B-factor overall	115	92	--	144	120	--
R.m.s. deviations						
Bond lengths (Å)	0.006	0.005	--	0.003	0.004	--
Bond angles (°)	0.606	0.563	--	0.632	0.545	--
**Validation**						
Molprobity clashscore	12.57	11.81	--	14.11	13.43	--
Rotamer outliers (%)	5.4	5.9	--	1.0	5.8	--
C_β deviations (%)	0.0	0.0	--	0.0	0.0	--
Ramachandran plot						
Favored (%)	95.2	95.7	--	96.6	95.0	--
Allowed (%)	4.8	4.3	--	3.4	5.0	--
Outliers (%)	0.0	0.0	--	0.0	0.0	--

This table notes the microscope collection parameters, as well as the map and model values used for all the final reconstructions and atomic models.

Movie Legends

Video S1. Example full field of view from TIRF assays of E254N MTs growing in the presence of EB3, related to Fig. 2. mGFP-EB3 signal (green) shows heterogeneous binding patterns on nonfluorescent E254N MTs polymerizing from CF640R-labeled GMPCPP-stabilized MT “seeds” (magenta). E254N tubulin 12.5 μM, mGFP-EB3 20 nM. Time stamp indicates min:sec. Scale bar 10 μm.

Video S2. Two nonfluorescent E254N MTs growing from CF640R-labeled GMPCPP-stabilized MT “seeds” (magenta) in the presence of 40 nM mGFP-EB3 (green). Each MT exhibits relatively uniform EB3 binding. The relative binding of mGFP-EB3 indicates a stronger affinity on the lattice of the microtubule on the left. E254N tubulin 12.5 μM. Time stamp indicates min:sec. Scale bar 5 μm.

Video S3. E254N microtubules polymerizing from CF640R-labeled GMPCPP-stabilized MT “seeds” (magenta) from 3 separate experiments exhibiting different behaviours, after transitioning to the “bright” state at the growing tip (see Fig. 2F for quantification). mGFP-EB3 is shown in green. Top: MT in the presence of 5 nM EB3 undergoing the “bright to dim” transition in the lattice. Middle: MT in the presence of 20 nM EB3 showing a “stable boundary” between dim and bright segments. Bottom: MT in the presence of 40 nM EB3 undergoing the “dim to bright” transition in the lattice. E254N tubulin 12.5 μM. Time stamp indicates min:sec. Scale bar 5 μm.

Video S4. Morph between E254N MTs and wildtype MTs showing the rotation of pfs proximal to the seam.

SI References

1. J. Roostalu *et al.*, The speed of GTP hydrolysis determines GTP cap size and controls microtubule stability. *Elife* **9**, e51992 (2020).
2. A. D. Cook, S. W. Manka, S. Wang, C. A. Moores, J. Atherton, A microtubule RELION-based pipeline for cryo-EM image processing. *J Struct Biol* **209**, 107402 (2020).
3. R. Zhang, E. Nogales, A new protocol to accurately determine microtubule lattice seam location. *J Struct Biol* **192**, 245-254 (2015).
4. R. Zhang, G. M. Alushin, A. Brown, E. Nogales, Mechanistic Origin of Microtubule Dynamic Instability and Its Modulation by EB Proteins. *Cell* **162**, 849-859 (2015).
