# Supplementary figures and images for: Structural transitions in the GTP cap visualized by cryo-electron microscopy of catalytically inactive microtubules
Source: Proc Natl Acad Sci U S A. 2022 Jan 7;119(2):e2114994119. doi: 10.1073/pnas.2114994119 (PMC8764682; doi:10.1073/pnas.2114994119)

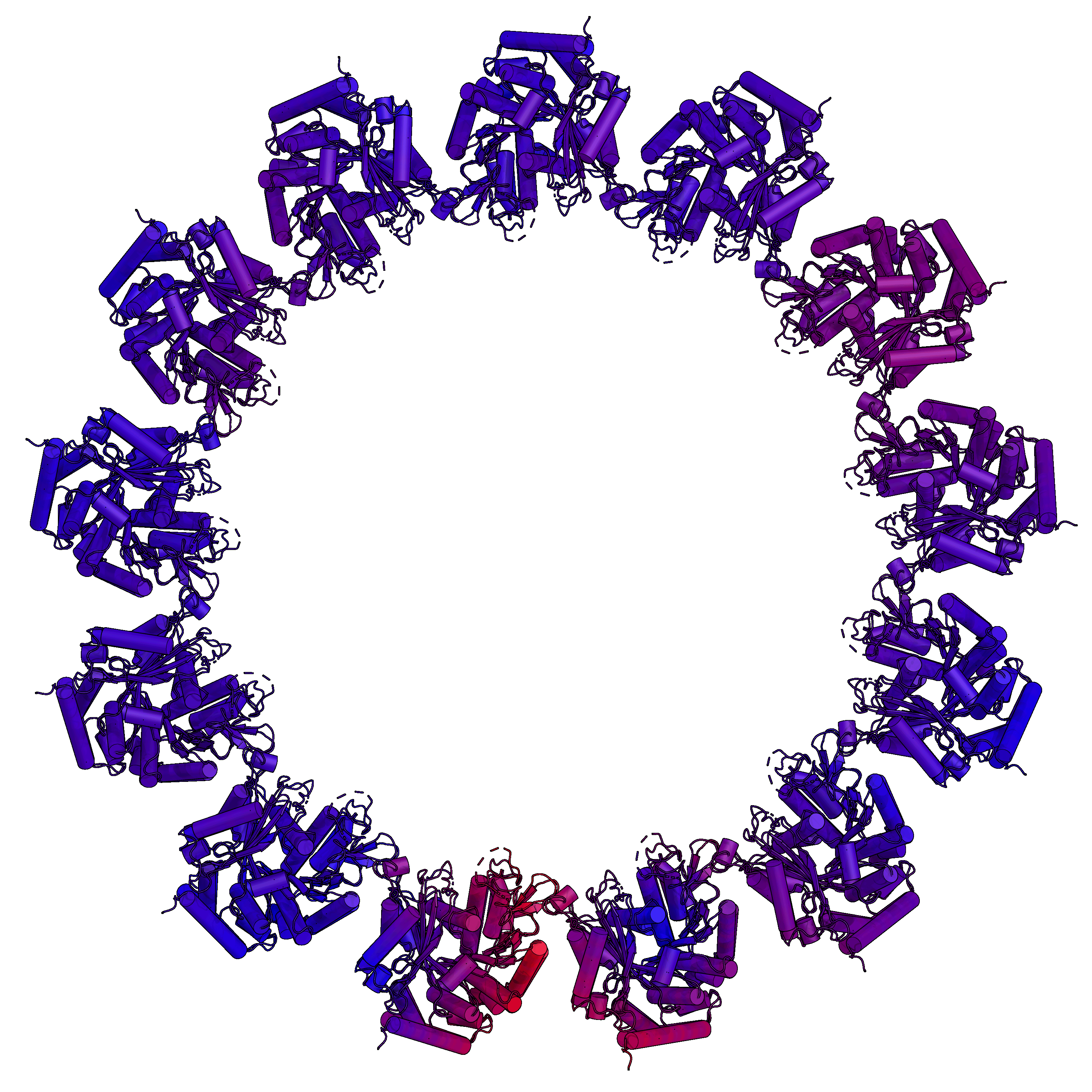

Supplement: Supplementary File [file pnas.2114994119.sm04.gif]
